# Development of an in vitro model of acquired resistance to toceranib phosphate (Palladia®) in canine mast cell tumor

**DOI:** 10.1186/1746-6148-10-105

**Published:** 2014-05-06

**Authors:** Charles HC Halsey, Daniel L Gustafson, Barbara J Rose, Amber Wolf-Ringwall, Robert C Burnett, Dawn L Duval, Anne C Avery, Douglas H Thamm

**Affiliations:** 1 Program in Cell and Molecular Biology, Colorado State University, Fort Collins, CO, USA; 2 Department of Microbiology, Immunology, and Pathology, Colorado State University, College of Veterinary Medicine and Biomedical Sciences, Fort Collins, CO, USA; 3 Department of Clinical Sciences, Colorado State University, College of Veterinary Medicine and Biomedical Sciences, Fort Collins, CO, USA; 4 Comprehensive Cancer Center, University of Colorado, Aurora, CO, USA; 5 Laboratory of Cancer Biology and Genetics, Center for Cancer Research National Cancer Institute, NIH, Bethesda, MD, USA

**Keywords:** Canine mast cell tumor, Toceranib, *c-kit*, Acquired resistance

## Abstract

**Background:**

Mast cell tumors (MCTs) are the most common skin tumors in dogs and exhibit variable biologic behavior. Mutations in the *c-kit* proto-oncogene are associated with the tumorigenesis of MCTs, resulting in growth factor-independent and constitutive phosphorylation of the KIT receptor tyrosine kinase (RTK). Toceranib (TOC) phosphate (Palladia**®**) is a KIT RTK inhibitor that has biological activity against MCTs. Despite these benefits, patients ultimately develop resistance to TOC. Therefore, there is a need to identify distinguishing clinical and molecular features of resistance in this population.

**Results:**

The canine C2 mastocytoma cell line contains an activating mutation in *c-kit*. Three TOC-resistant C2 sublines (TR1, TR2, TR3) were established over seven months by growing cells in increasing concentrations of TOC. TOC inhibited KIT phosphorylation and cell proliferation in a dose-dependent manner in the treatment-naïve, parental C2 line (IC_50_ < 10 nM). In contrast, the three sublines were resistant to growth inhibition by TOC (IC_50_ > 1,000 nM) and phosphorylation of the KIT receptor was less inhibited compared to the TOC-sensitive C2 cells. Interestingly, sensitivity to three structurally distinct KIT RTK inhibitors was variable among the sublines, and all 3 sublines retained sensitivity to the cytotoxic agents vinblastine and lomustine. Sequencing of *c-kit* revealed secondary mutations in the juxtamembrane and tyrosine kinase domains of the resistant sublines. These included point mutations in TR1 (Q574R, M835T), TR2 (K724R), and TR3 (K580R, R584G, A620S). Additionally, chronic TOC exposure resulted in *c-kit* mRNA and KIT protein overexpression in the TOC-resistant sublines compared to the parental line. C2, TR1, TR2, and TR3 cells demonstrated minimal P-glycoprotein (P-gp) activity and no functional P-gp.

**Conclusions:**

This study demonstrates the development of an *in vitro* model of acquired resistance to targeted therapy in canine MCTs harboring a *c-kit*-activating mutation. This model may be used to investigate the molecular basis of and strategies to overcome TOC resistance.

## Background

The increased understanding of molecular mechanisms driving tumorigenesis in a wide array of neoplasms has led to the development of novel targeted therapies. While tyrosine kinase inhibitors (TKIs) are routinely employed in human oncology with success, their use in veterinary medicine is limited. Two small molecule tyrosine kinase inhibitors, toceranib (TOC) phosphate (Palladia®; Zoetis, Madison, NJ) and masitinib (Masivet®, Kinavet®; AB Science, Paris, France) have been approved by the FDA for use in veterinary medicine for the treatment of recurrent, non-resectable intermediate and high grade canine cutaneous mast cell tumors (MCTs) [1]. The success of targeted therapies in both human and veterinary oncology, however, is largely tempered by the inevitable development of drug resistance.

Cutaneous MCTs are the most common skin tumors in dogs, accounting for up to 21% of all canine cutaneous tumors, and exhibit variable biologic behavior [2-4]. Cutaneous MCTs commonly present as a solitary mass in older dogs with a mean age of onset of 9 years old. There is no sex predilection. While all breeds can be affected, Boxers, Boston terriers, Labrador retrievers, Weimaraners, Bulldogs, Beagles, and Schnauzers are over-represented [5].

Activating mutations in the juxtamembrane, kinase and ligand binding domains of the *c-kit* proto-oncogene have been associated with the tumorigenesis of canine MCTs, resulting in growth factor-independent and constitutive phosphorylation of the KIT receptor tyrosine kinase (RTK). Approximately one-third of canine MCTs carry a *c-kit* mutation and the majority of MCTs with *c-kit* mutations are histologically intermediate or high grade [2,6,7]. While the majority of gain-of-function mutations of *c-kit* have been identified in exon 11 of canine MCTs, exons 8 and 9, and less commonly exon 17, also acquire activating mutations [8,9]. Our laboratory and others have shown that *c-kit* mutations, particularly internal tandem duplications (ITD) in the juxtamembrane domain, are significantly associated with an increased incidence of recurrent disease, metastasis, and death [2,6-8,10-12]. As such, small molecule inhibitors of KIT are an attractive therapeutic strategy for MCTs in dogs.

Toceranib phosphate is one such receptor tyrosine kinase inhibitor of KIT, approved for the treatment of recurrent, non-resectable grades 2 and 3 canine MCTs [13,14]. While TOC has demonstrated significant biological activity, its usefulness is significantly limited by the eventual acquisition of drug resistance. In a multi-center, placebo-controlled, double-blind, randomized study of oral TOC, approximately 40% of dogs experienced an objective response while the remaining 60% demonstrated no response, likely due to *de novo* resistance. Two-thirds of the responders were positive for an activating mutation in *c-kit*. The average time to tumor progression in all responders was 18 weeks; therefore, virtually all dogs with MCTs have either intrinsic TOC resistance or eventually develop resistance [13]. Therefore, there exists a need to identify distinctive clinical and molecular features of resistance in this population.

The aim of the current study was to develop a model of acquired TOC resistance in canine MCT. Acquired resistance was modeled *in vitro* using the TOC-sensitive C2 canine MCT cell line to subsequently allow us to investigate mechanisms of acquired resistance in order to ultimately develop second-line inhibitors as well as rational drug combination therapies for the treatment of TOC-resistant MCTs in dogs.

## Results

### Toceranib-resistant C2 cells emerged during chronic, stepwise TOC treatment

**Figure 1:**
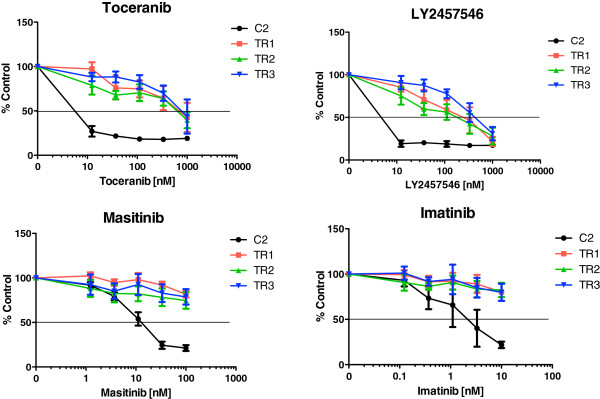
Dose-dependent growth inhibition of parental line (C2) and three resistant sublines (TR1, TR2, TR3) after incubation with increasing concentrations of toceranib phosphate or three other KIT receptor tyrosine kinase inhibitors (LY2457546, masitinib, imatinib) for 72 hours.

**Figure 2:**
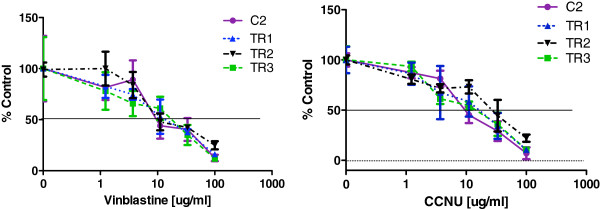
Dose-dependent growth inhibition of parental line (C2) and three resistant sublines (TR1, TR2, TR3) after incubation with increasing concentrations of vinblastine or CCNU (lomustine) for 72 hours.

To explore mechanisms of acquired TOC resistance in canine MCT, we generated three resistant sublines from the TOC-sensitive exon 11 ITD *c-kit* mutant C2 cell line designated TR1, TR2, and TR3. Growth of the parental C2 cells was inhibited by TOC in a dose-dependent manner with an IC_50_ of <10 nM. In contrast, TR1, TR2, and TR3 sublines were resistant to inhibition by TOC (IC_50_ > 1,000 nM) (Figure 1). Sensitivity to three other KIT RTK inhibitors was similar to the observed resistance to TOC. The parental line as well as all three sublines retained sensitivity to the cytotoxic agents vinblastine (VBL) and CCNU (Figure 2). Following 72 hr culture in the presence of increasing concentrations of TOC, treatment naïve, parental C2 cells detached from the culture flask and became rounded, shrunken, and clumped with increased exposure to TOC. In contrast, TOC-induced morphologic differences were not identified in the resistant sublines.

### Toceranib induces apoptosis in parental C2 cells, but not the TOC-resistant sublines

**Figure 3:**
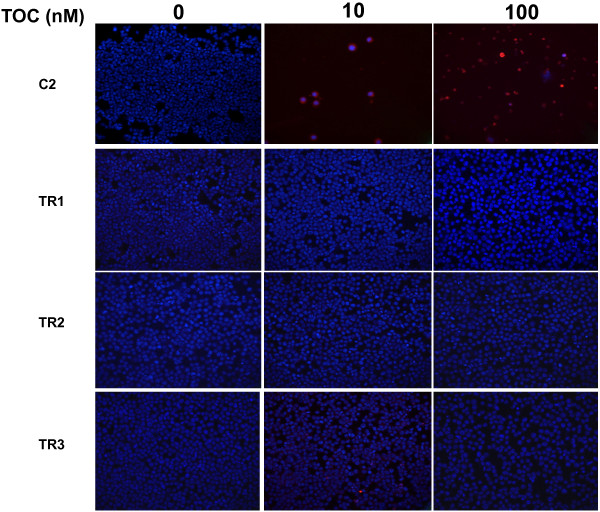
Effect of toceranib and vinblastine (B) on the induction of apoptosis in C2, TR1, TR2, and TR3 cells; Red- TUNEL; DAPI counterstain.

**Figure 4:**
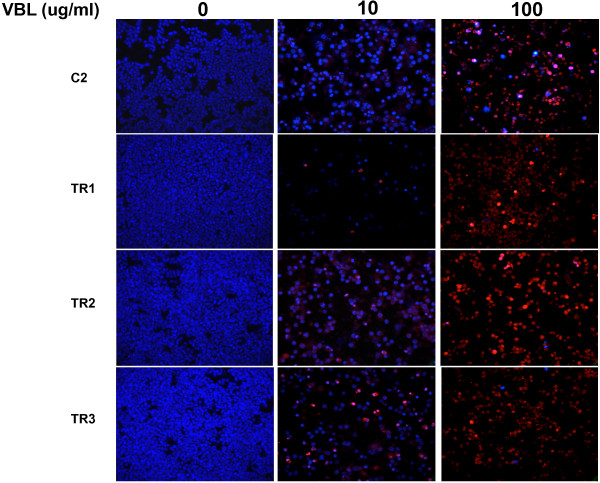
Effect of vinblastine on the induction of apoptosis in C2, TR1, TR2, and TR3 cells; Red- TUNEL; DAPI counterstain.

Tyrosine kinase inhibitors have been shown to promote growth inhibition in C2 cells by induction of apoptosis and cell-cycle arrest [15]. To explore this, Terminal Deoxynucleotidyltransferase-Mediated dUTP Nick End Labeling (TUNEL) assays and morphological evaluations were performed on all four cell lines to determine the effects of TOC and the cytotoxic agents, VBL and CCNU, on apoptosis. Following 72 hr of increasing exposure to TOC, a qualitative increase in the number of cells displaying increased TUNEL reactivity and morphologic evidence of apoptosis (chromatin condensation and nuclear fragmentation) was observed in the parental line. In contrast, no increase in either positive TUNEL staining or morphologic evidence of apoptosis was observed in the three TOC-resistant sublines (Figure 3). The parental line and all three resistant sublines demonstrated an equivalent increase in both TUNEL staining and apoptotic morphology after 72 hr of VBL (Figure 4) or CCNU exposure (data not shown).

### KIT phosphorylation in resistant cells does not decrease after toceranib treatment

**Figure 5:**
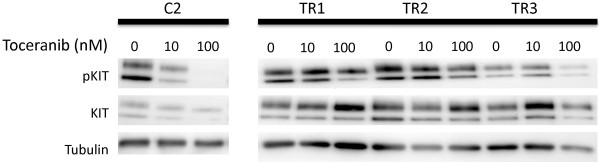
Western blot analysis of KIT activation (phosphorylated KIT) in parental line (C2) and three resistant sublines (TR1, TR2, TR3) after incubation with increasing concentrations of toceranib phosphate for 24 hours.

To determine whether the lack of growth inhibition observed in the resistant sublines in Figure 1A was due to a lack of inhibition of autophosphorylation by TOC, the cells were incubated with increasing concentrations of TOC for 24 hours and western analysis for phosphorylated and total KIT was performed. TOC inhibited KIT phosphorylation in the parental C2 line in a dose-dependent manner while phosphorylation of the KIT receptor was maintained in the presence of TOC in all three resistant sublines (Figure 5).

### Chronic TOC exposure resulted in significant overexpression of c-kit mRNA and KIT protein in the TOC-resistant sublines

**Figure 6:**
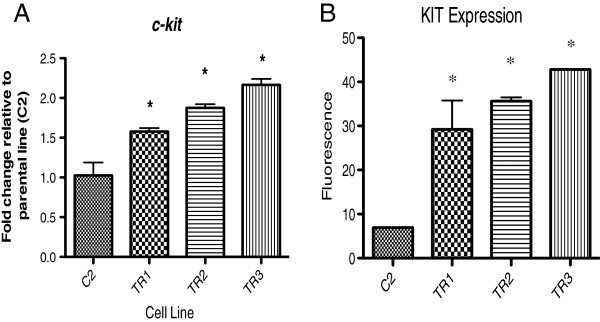
**Analysis of *c-kit *** **and KIT expression in C2, TR1, TR2, and TR3 cells by RT-qPCR (A) and flow cytometry (B), respectively.**

To investigate whether overexpression of the target kinase contributes to the observed TOC resistance, *c-kit* mRNA and KIT protein expression was measured by real-time quantitative PCR and flow cytometry, respectively. Indeed, TOC-resistant sublines demonstrated up to a four-fold increase in KIT receptor expression compared to the parental, treatment naïve C2 cells (Figure 6A and B). Additionally, densitometric analysis of chemiluminescent signals of total KIT from Figure 5 was performed using Image J software (National Institutes of Health, Bethesda, MD), which demonstrated significant overexpression of KIT in all three resistant sublines compared to the parental line (data not shown).

### C2, TR1, TR2, and TR3 sublines demonstrate minimal P-gp activity and no functional P-gp

**Figure 7:**
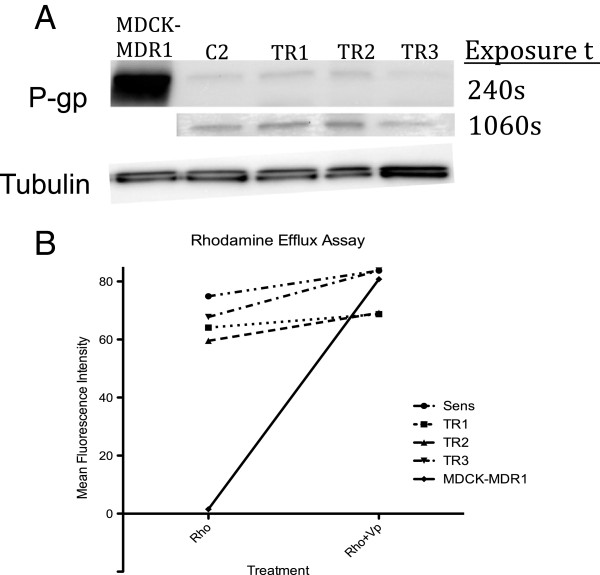
**Expression and function of P-gp in C2, TR1, TR2, and TR3 cells (A) Western blot analysis of P-gp expression in at 240 and 1060 second exposures and (B) Rhodamine efflux/uptake assay in the same cells as A.** Administration of the P-gp inhibitor, verapamil, increases fluorescence signal in lines with functional P-gp (MDCK-MDR1) with relatively no change in signal in lines without functional P-gp (C2, TR1, TR2, TR3).

To determine if TOC resistance is caused by overexpression and increased functional activity of the drug transporter P-glycoprotein (P-gp), western analysis and rhodamine uptake/efflux assays were performed, respectively, in all four sublines. While MDR1-overexpressing MDCK cells showed significant overexpression of P-gp, all four sublines demonstrated little to no P-gp expression, even when blots were overexposed (Figure 7A). Furthermore, densitometric analysis of chemiluminescent signals of P-gp showed no significant differences in P-gp expression in the resistant sublines versus the parental line (data not shown). The activity of P-gp in the same cells was determined by rhodamine uptake/efflux. As expected, MDR1-MDCK cells demonstrated a lower fluorescence signal compared to C2, TR1, TR2, and TR3 cells. Administration of the P-gp inhibitor, verapamil, increased the fluorescence signal in the MDR1-MDCK cells, however, no shift in the fluorescence signal was detected in the C2, TR1, TR2, and TR3 cells (Figure 7B).

### Secondary c-kit mutations are present in the juxtamembrane and kinase domains of c-kit in resistant sublines

**Figure 8:**
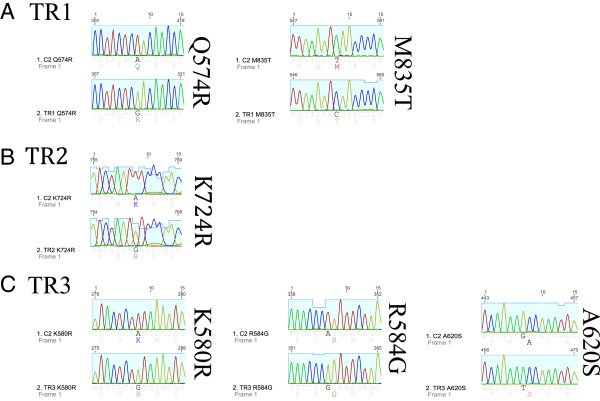
**Point mutations identified in 7-10 clones of full-length *c-kit *from (A) TR1, (B) TR2, and (C) TR3 sublines.** Mutations were commonly identified in functional domains of the KIT receptor.

To assess whether the development of secondary mutations in the *c-kit* gene conferred the observed resistance to TOC, full-length canine *c-kit* from the TOC-sensitive and -resistant sublines was cloned and sequenced. cDNA sequence analyses of full length *c-kit* from each clone after assembly and comparison of 7-10 clones from each subline was performed. A total of six point mutations were identified in the juxtamembrane and kinase domains of 30-50% of the resistant clones. These included Q574R in exon 11 and M835T in exon 18 of TR1; K724R in exon 15 of TR2; and K58R in exon 11, R584G in exon 11, and A620S in exon 12 of TR3 (Figure 8). These novel mutations were not identified in any of the parental C2 clones. Additionally, alternative splice sites between exons 9 and 10 and exons 17 and 18 were identified in all sublines. These transcripts utilize alternate splice donors (GT) 3′ to exons 9 and 17. Furthermore, retention of the original 48-bp internal tandem duplication in exon 11 of the parental line was observed in all three resistant sublines.

## Discussion

The identification of protein kinases as instrumental regulators in the tumorigenesis of many forms of neoplasia has led to the development of numerous small molecule kinase inhibitors for the treatment of cancer. The understanding of the molecular pathway driving the development of at least some canine cutaneous MCT, its addiction to a dominant oncogene, coupled with the identification of a “druggable” target has resulted in significant progress toward its treatment. Activating mutations in the *c-kit* proto-oncogene confer growth-factor independent activation of the KIT receptor tyrosine kinase, subsequent downstream signaling, and enhanced proliferation and survival of malignant mast cells [4,16]. Ligand-independent activation of the KIT pathway most commonly occurs due to a mutation in the juxtamembrane domain in exon 11 [6]. This domain has a negative regulatory function by maintaining the KIT receptor in its inactive conformation in the absence of ligand binding. Mutations in this domain result in an active conformation due to disruption of the inhibitory motif resulting in autophosphorylation of the KIT receptor and downstream signaling [17]. Upon binding the ATP-binding pocket within the TK domain, the small molecule KIT receptor tyrosine kinase inhibitors abrogate KIT signaling and induce growth inhibition and apoptosis [14,18]. Dogs with MCTs harboring an activating mutation in the *c-kit* proto-oncogene have demonstrated significantly increased response rates to TOC [13]. Despite these benefits, the responses are often transitory as tumors commonly develop resistance to TOC.

To begin to identify mechanisms of acquired resistance to TOC, we have successfully developed a model of acquired resistance using a canine MCT cell line by continuously exposing cells to increasing concentrations of TOC, resulting in three independent sublines that are resistant to TOC. The C2 MCT cell line harbors the KIT-activating ITD mutation in exon 11, which represents the most common mutation in canine MCT [8,10]. In one study, 64% of KIT mutations identified in canine MCT were ITDs in exon 11 [8]. As such, the C2 cell line is a clinically-relevant canine MCT line for these investigations. While the parental C2 line demonstrated dose-dependent growth inhibition following treatment with TOC, the three sublines, TR1, TR2, and TR3, remained resistant to TOC exposure. Similarly, TOC exposure caused an induction of apoptosis in the parental line while no evidence of apoptosis was observed in the three sublines following similar TOC exposure. Importantly, the TOC-resistant sublines retained sensitivity to the cytotoxic agents vinblastine and CCNU, and demonstrated variable sensitivity to other KIT kinase inhibitors. This lack of apparent cross-resistance to the conventional cytotoxic agents VBL and CCNU suggests that these drugs may remain active in patients with TOC-refractory disease. In dogs, the range of achievable concentrations for TOC in vivo has been reported between 30 nM (Cmin) and 200 nM (Cmax) [19,20]. In the current study, growth inhibition assays were carried out to 1 uM, nearly 5-fold the reported Cmax for TOC. However, there may be a small therapeutic window in which higher concentrations of masitinib may override TOC-resistance in MCT as the tested concentrations were lower than the Cmax reported in dogs (1.3 uM-1.5 uM) but, this is beyond the scope of the current investigations [21].

There are several reported pathway-dependent mechanisms of acquired resistance to tyrosine kinase inhibitors. One of the most common mechanisms is acquisition of secondary mutations within the target oncogene leading to either reactivation of the target protein or induction of a conformational change in the drug binding pocket resulting in reduced binding affinity [22-26]. As KIT tyrosine kinase inhibitors bind to the ATP-binding pocket of a kinase in a competitive fashion, mutations located in the in the drug/ATP-binding pocket of the receptor are associated with acquired drug resistance. Heinrich and co-workers showed that secondary point mutations located in the ATP-binding pocket of the KIT receptor (encoded by exons 13 and 14) are associated with resistance to imatinib, a KIT receptor tyrosine kinase inhibitor, in gastrointestinal stromal tumors (GISTs) [27]. In the current study, the observed variable resistance to the three other KIT inhibitors, both between the three inhibitors (imatinib, masitinib and LY2457546) and among the three resistant sublines, suggests that there may be differences in drug binding kinetics among the four compounds and perhaps differences in mechanisms of acquired resistance between the three sublines, respectively.

Engagement of alternative or bypass signaling pathways is another common mechanism of acquired resistance to receptor tyrosine kinase inhibitors [28-31]. This can occur independent of the target oncogene to which a tumor is addicted. Indeed, the activation of a bypass pathway has been shown to overcome KIT inhibition in human GISTs. GIST cells resistant to imatinib demonstrated increased activation of the AKT pathway leading to continued cell growth and survival [32,33]. Nazarian and co-workers showed that melanomas harboring a BRAF (V600E) mutation eventually become resistant to the RAF-selective inhibitor, PLX4032, by activation of an alternative survival pathway mediated by PDGFRβ [34].

In addition to secondary mutations in the target oncogene and activation of bypass signaling pathways, resistance to targeted therapies can also occur through activation of effector proteins upstream and/or downstream of the intended target. Nazarian and co-workers also demonstrated reactivation of NRAS signaling in PLX4032-refractory melanomas leading to MAPK pathway reactivation and disease progression [34]. Similarly, Wagle and co-workers demonstrated activating mutations in MEK1 and subsequent reactivation of the MAPK pathway following treatment of melanoma with PLX40 [35].

To begin to explore these possibilities in our TOC-resistant canine MCT model, we examined KIT activation status in the parental and TOC-resistant C2 sublines by western blot analysis using an antibody against KIT phosphorylated at Tyr719. While phosphorylated KIT was reduced in a dose-dependent manner in the parental line, KIT activation was maintained in the presence of increasing concentrations of TOC in all three resistant sublines. These data led to the hypothesis that acquisition of a secondary mutation in the *c-kit* proto-oncogene would be, in part, responsible for the observed TOC resistance. To that end, full-length canine *c-kit* was cloned and sequenced. The original ITD mutation in exon 11 was maintained in all three resistant sublines. Indeed, we detected several different point mutations in the resistant sublines leading to amino acid substitutions. Interestingly, all of these mutations were located in the functional domains of the KIT receptor. Computational modeling of these mutations is in process to ascertain whether they impede contact between the KIT inhibitors, including TOC, and their binding sites or alter spatial conformation of the target protein. The frequency with which these mutations were identified in the resistant clones was between 30-50%. This likely represents the heterogeneity associated with resistance mechanisms. It has been shown that multiple drug-resistant mutations and disparate mechanisms of resistance can frequently occur in a single population of tumor cells [30,36-38]. These include from multiple secondary mutations in the target kinase as well as independent mechanisms such as activation of a bypass pathway. Other single nucleotide polymorphisms (SNPs) were identified in single clones. These are likely a result of polymerase error when observed in a single clone, but when duplicated represent transcript heterogeneity resulting from genomic instability perhaps as a result of deficiencies in the DNA repair machinery. Alternative signaling pathways that bypass inhibition of the target protein, KIT, were not pursued in the current study. Constitutive activation of KIT in the presence of TOC in the resistant sublines strongly suggests that the mechanism of resistance occurs at the level of the KIT receptor.

A final pathway-dependent mechanism of acquired resistance is through genomic amplification of the target gene. Amplification of the target gene and subsequent overexpression of the target kinase, can alter the drug-target stoichiometry such that inhibition is diminished and cell survival and proliferation persists [39-43]. Genomic amplification of *c-kit* has been reported in imatinib-resistant GISTs as a mechanism of acquired resistance [44]. Ercan and co-workers demonstrated that although non-small lung cancers harboring a *EGFR* T790M mutation transiently respond to EGFR inhibitors, clones over-expressing *EGFR* T790M eventually emerge leading to clinical resistance [41]. Increased expression of the target protein BCR/ABL, resulting from genomic oncogene amplification, was observed in chronic myelogenous leukemia (CML) cell lines that became refractory to the selective ABL tyrosine kinase inhibitor imatinib [40]. In the current study, analysis of *c-kit* mRNA expression by real-time quantitative PCR demonstrated a significant increase in *c-kit* expression in the TOC-resistant C2 sublines compared to the treatment-naïve parental C2 cells. To determine if this increase in *c-kit* transcript led to a subsequent increase in KIT receptor expression, flow cytometry was performed. Indeed, a significant increase in KIT receptor expression was demonstrated in the three TOC-resistant C2 sublines compared to the TOC-sensitive parental C2 cells. This could confer the observed resistance as binding of TOC to the overexpressed target could deplete the amount of intracellular drug available. As such, increasing the dose of TOC would be a reasonable therapeutic approach to overcome KIT-overpexpressing TOC-resistant canine mast cell tumors. However, in the current model, growth inhibition assays were carried out to doses of TOC 100-fold the IC_50_ of the treatment naïve parental C2 cells and an IC_50_ was not reached in all three resistant lines. Therefore, these data suggest that a four-fold increase in expression of the target protein by itself is likely not adequate to confer the observed resistance. Amplification of the target oncogene, and subsequent overexpression of the encoded target protein, may have been driven in response to continued pressure by the KIT inhibitor. Alternatively, because the TOC-resistant sublines initially responded to TOC and maintained the original ITD activating *c-kit* mutation in exon 11, it is possible that the resistant sublines were derived from a distinct *c-kit*-amplified subpopulation of *c-kit*-mutant cells that were subsequently selected for during TOC administration.

While the current study focuses on pathway-dependent mechanisms of KIT RTK resistance, there are several reported pathway-independent mechanisms of resistance that were investigated. These include pharmacological factors that ultimately diminish drug exposure. Drug-efflux pumps, such as P-glycoprotein (P-gp) encoded by *MDR1*, have been shown to be overexpressed in several TKI-resistant tumors and cell lines. Mahone and co-workers reported a significant overexpression of P-gp in imatinib-resistant leukemia cell lines [45]. Furthermore, sensitivity was restored following administration of several P-gp inhibitors. Nakaichi and co-workers reported the expression of P-gp and *MDR-1* by western blot analysis and RT-PCR, respectively, in several canine MCT cell lines, excluding C2 [46]. Sunitinib, a structural analog of toceranib, has been shown to be a substrate of P-gp. As such, we investigated the role of drug efflux in TOC-resistant canine C2 cells as a mechanism of resistance by measuring P-gp expression and function [47]. The expression of P-gp was determined in all four C2 sublines and MDR1-overexpressing MDCK cells by western analysis. While the presence of P-gp was confirmed in all four sublines, there were no significant differences in expression between the TOC-sensitive cells and TOC-resistant cells. Furthermore, all four C2 sublines showed minimal functional P-gp as measured by rhodamine efflux with or without administration of the P-gp inhibitor, verapamil.

## Conclusions

Sustained KIT signaling appears required for *c-*kit mutant MCT survival. Regardless of the specific mechanism of acquired TOC resistance outlined above, all may lead to reactivation of the KIT signaling pathway and ultimately tumor progression. Our results demonstrate that continuous, chronic exposure of C2 cells to TOC causes eventual drug resistance. We demonstrate that overexpression of the KIT receptor is, in part, responsible for the observed TOC resistance, and have identified several candidate mutations that may play a role in resistance acquisition. The identification of these and other potential mechanisms of TOC resistance is necessary for the identification of second line KIT inhibitors or alternate therapeutic strategies for the treatment of high grade, non-resectable canine MCT that are refractory to TOC. Furthermore, we have created *in vitro* tools that can be utilized for future study of re-sensitization strategies for TOC-resistant canine MCT.

## Methods

### Cell culture and generation of toceranib-resistant sublines from C2 cells

Toceranib phosphate was provided by Zoetis (Florham Park, NJ). Masitinib (AB1010, Kinavet®) and LY2457546 were provided by AB Science (Paris, France) and Elanco (Greenfield, IN), respectively. Imatinib was purchased from Selleck Chemical (Houston, TX). Vinblastine (VBL) and lomustine (CCNU) were purchased from Sigma (St. Louis, MO). Stock solutions of all drugs were prepared in DMSO and stored at -20°C. The *c-kit* mutant canine C2 mastocytoma cell line, derived from a spontaneously occurring cutaneous MCT, was used as the parental cell line [48]. Cells were propagated in RPMI 1640 supplemented with 2 mM L-glutamine, 10% FBS, 100 g/mL streptomycin, and 100 U/mL penicillin in a 37°C incubator under a humidified atmosphere of 5% CO_2_. TOC-resistant C2 cells were selected by growing C2 cells in concentrations of TOC ranging from 0.02 uM to 0.3 uM and increasing in 0.025-0.05 uM increments. Three independent, TOC-resistant sublines were established over a period of 7 months.

### Drug sensitivity assays

The sensitivity and resistance of each cell line to TOC, three other kinase inhibitors, and the cytotoxic agents VBL or CCNU were determined by measuring relative viable cell number using a fluorometric bioreductive assay (Alamar Blue, Promega; Madison, WI). All three C2 sublines as well as the treatment naïve, parental C2 cells were plated in triplicate in 96-well plates at densities of 2,000 cells per well. Cells were treated with increasing concentrations of TOC, three other KIT kinase inhibitors, VBL or CCNU for 72 hours. Alamar Blue reagent was added to all wells, plates were incubated for 1 hour at 37°C, and fluorescence was measured on a BioTek plate reader (BioTek, Winooski, VT). Dose-response curves were generated using Prism (GraphPad Software, La Jolla, CA).

### Terminal Deoxynucleotidyltransferase-Mediated dUTP Nick End Labeling (TUNEL) apoptosis assays

To evaluate drug effects on the induction of apoptosis, the C2 parental and three TOC-resistant sublines were treated for 24 hr with increasing concentrations of TOC (0-100 nM) and the cytotoxic agents VBL and CCNU (0 ug/mL-100 ug/mL). Cells were harvested and resuspended in media. Approximately 250,000 cells were centrifuged at 40 x g for 4 minutes onto glass slides. Cytospins were dried and stored at 4°C overnight followed by fixation in 4% paraformaldehyde for 60 min at room temperature. Cells were permeabilized with 0.1% Triton X-100 in 0.1% sodium citrate solution for 2 min on ice. TUNEL staining was carried out following the manufacturer’s instructions (Roche Applied Science, Indianapolis, IN). Slides were counterstained and mounted with DAPI (Vector, Burlingame, CA). Image analysis was performed using AxioVision 4.3 system software from Carl Zeiss using an Axioplan 2 imaging scope coupled with an AxioCam HRc Carl Zeiss camera.

### Western blot analysis

To evaluate drug effects on KIT autophosphorylation, parental C2 cells and the resistant sublines were incubated for 24 hours with increasing concentrations of TOC (0-100 nM) and phosphorylated and total KIT were analyzed by western blot. Cells were resuspended in lysis buffer containing 1% Triton X-100, 100 nM sodium orthovanadate, 0.2 mM PMSF, 1 M Tris, 1 M NaCl, and 7X protease inhibitor cocktail (Roche Applied Science, Indianapolis, IN), incubated on ice for 15 min, and centrifuged for 5 min. Protein was separated by SDS-PAGE on a 6% acrylamide gel and transferred onto a polyvinylidene difluoride membrane. Membranes were blocked for 60 min at room temperature in 5% bovine serum albumin. Immunolabeling for KIT was performed using a rabbit polyclonal anti-human antibody (Dako, Carpinteria, CA) at 1:1000 while immunolabeling for pKIT was performed using a rabbit polyclonal anti-human antibody (Cell Signaling Technology, Beverly, MA) at 1:2000 for 16 hours at 4°C, followed by incubation with HRP-conjugated anti-rabbit antibody at 1:5000 for 30 min at room temperature. Immunoreactive bands were detected using enhanced chemiluminescent reagents (Thermo Scientific, Rockford, IL).

### Mutational analysis: cloning and sequencing of c-kit

**Figure 9:**
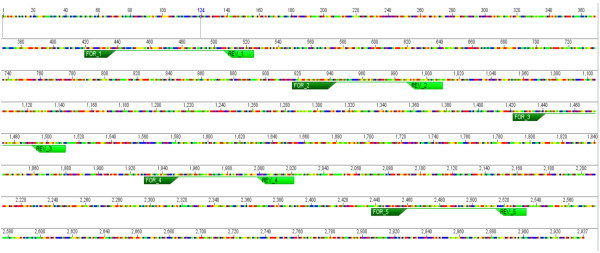
**Sequencing strategy of full-length canine *c-kit *** **with forward and reverse internal sequencing primers.**

Full-length canine *c-kit* from the TOC-sensitive and -resistant sublines was cloned and sequenced. Total RNA was extracted from C2 cells using RNeasy Mini-kit after homogenization using QIA-shredder columns according to the manufacturers instructions (Qiagen; Valencia, CA). First strand cDNA was synthesized using ThermoScript™ RT-PCR System (Invitrogen; Carlsbad, CA) according to the manufacturers instructions. Full-length canine *c-kit* was amplified using Platinum® *Taq* DNA polymerase High Fidelity (Invitrogen) and the following primers: *c-kit* Forward AGGCTATCGCAGCCACCGCGATGAG and *c-kit* Reverse GATCGCTCTTGTTGGGGAGAC. The conditions for PCR amplification were as follows: pre-denaturation at 94°C for 2 min, 40 cycles of denaturation at 94°C for 30 sec, annealing at 57°C for 30 sec, extension at 68°C for 3 min 30 sec and, following the final cycle, an additional extension at 68°C for 7 min was performed. The PCR products were purified according to the QIAquick PCR purification kit instructions. The concentration was determined using a Nanodrop 1000 spectrophotometer (Thermoscientific; Wilmington, DE). The cDNA fragment of interest was ligated into a pGEM®-T easy vector (Promega) by T4 ligase at 4°C overnight. The product was transfected to competent DH5α bacteria. Positive recombinants were selected on a Luria-Bertani (LB) plate with X-gal and 100 μg/mL ampicillin. The white bacterial colonies were selected, amplified and plasmids were extracted and purified using the QIAquick DNA reagent kit (Qiagen). Positive clones were selected by restriction endonuclease digestion with *Eco*RI and SpeI restriction enzymes and positive recombinants were sequenced. Sequencing was performed using the dideoxynucleotide chain termination method (Sanger Method) with an automatic sequencer (ABI 3130xL Genetic Analyzer) using T7 and SP6 promoter primers and five internal sequencing primers (Table 1, Figure 9). Assembly, editing and comparison of all cDNA sequences was performed using Geneious Pro version 5.5.8 created by Biomatters (http://www.geneious.com/). Briefly, multiple clones from each cell line were compared to eliminate potential polymerase errors. For each clone, full-length *c-kit* sequence was assembled from a series of overlapping sequence reads. Contig assembly and multiple sequence alignments were performed using the “Assembly” and “Alignment” functions of Geneious, respectively.

**Table 1:** Forward and reverse sequencing primers

**Primer**	**Sequence range (bp)**	**Start site**	**5′-Sequence-3′**
FOR1	501–1000	448	GACGGACCCAGAAGTGACC
FOR2	1001–1500	948	CCTTGGAAGTAGTAGATAAAGGATTCA
FOR3	1501–2000	1453	AGTGGTTCAGAGTTCCATCG
FOR4	2001–2500	1948	CAAAGTCTTGAGTTACCTCGG
FOR5	2501–3000	2950	TGTGTGAAGCAGGAGGAGTG
REV1	500–1	552	CTGATCGTGATGCCAGCTT
REV2	1000–501	1040	CAATCAGATCCACATTCTGTCC
REV3	1500--1001	1542	GCAGAACTCCTGCCTACATTG
REV4	2000--1501	2040	TATTCTGTAATGACCAAGGTGGG
REV5	2500--2001	2552	TGAAAATGCTCTCAGGGGC

### c-kit and KIT expression

#### Real time-quantitative PCR (RT-qPCR)

To evaluate the effects of chronic TOC exposure on mRNA expression of *c-kit*, RT-qPCR was performed on both TOC-sensitive and –resistant C2 cells. RNA was extracted, purified, and cDNA synthesis performed as described above. RT-qPCR was performed on five biological replicates in triplicate with denaturation at 94°C for 2 minutes and 40 cycles of 30 seconds at 94°C (melting) and 60 seconds at 57°C and 3 minutes and 30 seconds at 68°C (annealing and elongation) followed by 7 minutes at 68°C using the iQ SYBR Green Supermix (Bio-Rad, Hercules, CA, USA) and 25 ng equivalent RNA input in 25 μL reactions on a Stratagene Mx3000P thermal cycler (Stratagene; La Jolla, CA). Primers were designed to be intron-spanning using Geneious. The standard curves, dissociation curves, and amplification data were collected using Mx3000P software and analyzed with the 2^(-ΔΔCt)^ method [49]. In all cases, the amplification efficiencies were greater than 90% and both amplicon size and sequence were confirmed. Expression levels were normalized to hypoxanthine phosphoribosyltransferase 1 (HPRT1) expression. HPRT was selected as a reference gene since it did not exhibit significant variation among our experimental samples. The *c-kit* forward primer sequence was 5′- TTGGTCTAGCCAGAGACATCAA -3′, the *c-kit* reverse primer sequence was 5′ TGAAAATGCTCTCAGGGGC -3′, the HPRT1 forward primer sequence was 5′-TGC TCG AGA TGT GAT CAA GG-3′ and the HPRT1 reverse primer sequence was 5′-TCC CCT GTT GAC TGG TCA TT-3′.

#### Flow cytometry

To evaluate the effects of chronic TOC exposure on KIT expression, flow cytometric analysis was performed on three biological replicates of TOC-sensitive and –resistant lines in triplicate. 250,000 parental C2 and TR1, TR2, and TR3 cells were incubated with 0.4ug PE-conjugated rat anti-mouse monoclonal CD117 (BD Pharmingen; San Jose, CA) for 30 minutes in the dark at room temperature, washed with 1X PBS, centrifuged at 200 x g for 5 minutes, and resuspended in 1X PBS. Data was acquired using a Gallios flow cytometer and Gallios software (Beckman Coulter; Brea, CA). Results were analyzed using Kaluza Analysis Software (Beckman Coulter). Cells were gated based on forward scatter and side scatter properties.

### P-gp expression/function

To evaluate the expression and function of P-gp in the TOC-sensitive and -resistant sublines, western blotting and rhodamine uptake/efflux was performed, respectively. C2, TR1, TR2, and TR3 cells were lysed as described above. As a positive control, MDR1-over-expressing canine Madin Darby Canine Kidney (MDCK) cells were used (kindly received from Dr. Michael Gottesmann, Laboratory of Cell Biology, National Cancer Institute, NIH, Bethesda, MD). Protein was separated by SDS-PAGE on a 6% acrylamide gel and transferred onto a polyvinylidene difluoride membrane. Membranes were blocked for 60 min at room temperature in 4% milk. Immunolabeling for MDR-1/Pg-p/ABCB1 was performed using a rabbit polyclonal anti-human antibody (Novus Biologicals, Littleton, CO) at 1:1000 followed by incubation with HRP-conjugated anti-rabbit antibody at 1:5000 for 30 min at room temperature. Immunoreactive bands were detected using enhanced chemiluminescent reagents (Thermo Scientific; Rockford, IL). Rhodamine uptake/efflux assays were performed as previously described [50,51]. Briefly, 200,000 cells were seeded in 6-well plates 24 hr prior to assay. Cells were incubated in rhodamine (3 μM) or rhodamine and verapamil (50 μg/mL) for 1 hr at 37°C. Rhodamine-containing media was removed, replaced with fresh media or media and verapamil, and placed at 37°C for 1 hr. Cells were harvested, washed, and flow cytometry was performed to measure fluorescence intensity.

### Ethical statement

This study does not need approval from an ethical committee.

## Abbreviations

MCT: Mast cell tumor; RTK: Receptor tyrosine kinase; TOC: Toceranib phosphate; TR: TOC-resistant; VBL: Vinblastine; CCNU: Lomustine; ITD: Internal tandem duplication; TUNEL: Terminal deoxynucleotidyl transferase dUTP nick end labeling; DAPI: 4′,6-diamidino-2-phenylindole; Q: Glutamine; R: Arginine; M: Methionine; T: Threonine; K: Lysine; G: Glycine; A: Alanine; S: Serine; P-gp: P-glycoprotein; MDR1-MDCK: Multi-drug resistant gene 1 Madin Darby canine kidney; ABCB1: ATP-binding cassette B1; GIST: Gastrointestinal stromal tumor; RPMI: Roswell Park Memorial Institute; FBS: Fetal bovine serum; PMSF: Phenylmethanesulfonylfluoride or phenylmethylsulfonyl fluoride; SDS-PAGE: Sodium dodecyl sulfate- Polyacrylamide gel electrophoresis; qRT-PCR: Real Time quantitative Reverse Transcription polymerase chain reaction; HPRT: Hypoxanthine-guanine phosphoribosyltransferase.

## Competing interests

Dr. Douglas Thamm is a paid consultant for Zoetis Animal Health.

## Authors’ contributions

CH drafted the manuscript, participated in study design, performed all western blot analyses, TUNEL assays, cloning and sequencing, quantitative real-time PCR, flow cytometric analysis, rhodamine efflux assays, and participated in all growth inhibition assays. DG participated in study design and P-gp expression and functional assays. BR established the resistant C2 sublines and participated in all growth inhibition assays. AWR participated in all growth inhibition assays. RB participated in study design and sequence alignment and analysis. DD participated in cloning and quantitative real-time PCR. AA participated in flow cytometric analysis. DT conceived the study, participated in study design, and helped draft the manuscript. All authors read and approved the final manuscript.
